# Strategy-Focused Agile Transformation: A Case Study

**DOI:** 10.1007/978-3-030-58858-8_17

**Published:** 2020-08-18

**Authors:** Helen Sharp, Katie Taylor

**Affiliations:** 6 IT University of Copenhagen, Copenhagen, Denmark; 7 University of British Columbia, Vancouver, BC Canada; 8 The Open University, Milton Keynes, MK7 6AA UK; 9 University Central Lancashire, Preston, UK

**Keywords:** Culture, Performance measurement, Strategic flexibility

## Abstract

Strategic agility enables an organisation to sense and seize opportunities, manage uncertainty and adapt to changes. This paper presents one case study of a traditional charitable organisation taking a strategy-focused approach to agile transformation. Interview data was collected over a 13-month period through interviews at different stages and with different members of the transformation team and Heads of Department. This case study illustrates the challenges faced in such a transformation, and shows that strategic agility requires different time horizons to co-exist: a future vision, a medium term set of objectives and a short term performance monitoring perspective.

## Introduction

The implementation of agility outside IT departments and across organisations, often referred to as enterprise agility, is growing in popularity, and is a significant challenge [4]. This is partly driven by the tensions that can arise when agile IT teams interact with non-agile departments in different parts of the organisation [6], and partly by the increasing need for organisations to be responsive to change [7]. Research in the managerial field refers to *flexibility* rather than *agility,* and although the similarities and differences are disputed, literature on flexibility provides a useful viewpoint for analysing enterprise agility. For example, Toni and Tonchia [13] identify four complementary dimensions of flexibility: economic, operational, organisational and strategic. The *economic* dimension has been addressed in conjunction with theories for management of financial buffers against demand uncertainties or external market shocks. The *operational* dimension deals with aspects of manufacturing system flexibility, e.g. ability to adapt the manufacturing system to different environmental conditions and a variety of product features. Agile software development literature [8] captures especially operational aspects related to software component development, e.g. management of rapidly changing business requirements and iterative delivery practices. The *organisational* dimension deals with models of organisation and labour flexibility in rapidly changing environments [13].

The *strategic* dimension may be viewed through culture [10], leadership [5] and dynamic capabilities [12] that enable an organisation to sense and seize opportunities, manage deep business uncertainty and adapt to changes in the business environment. From a strategic management perspective [12], strategy is not just a plan but a means to achieve agility through implementation of those plans, hence organisations achieve agility by forming an appropriate strategy and embedding the strategy vision, values, and goals at the operational level across the organisation.

Agile transformation of this kind is referred to using different names, including business agility and enterprise agility [6], but although process guidance for transformation can be found [1], empirical studies are lacking [3]. In particular, there is a lack of evidence illustrating how organisations transform to agility through a strategy focus, and the issues they encounter during this type of transformation. To address this, we conducted a 13-month case study during 2017 and 2018, to answer the research question: What issues arise within organisations focusing on strategic agility? This paper reports the findings of a case study [11], investigating a charitable organisation transforming to agility through a focus on *strategic agility*.

## Case Background

*“What is the role of a Victorian patriarchal provider of services for *<*disabled*>* people in an age where funding streams, public expectations, customer expectations, deem that we’re actually no longer relevant, fundamentally all of our lead indicators for the business are really unhealthy. Need to fundamentally transform and that makes it really big.”* Change manager (member of transformation team).

Our case organisation is a traditional charity for disabled people. It was originally two separate organisations with different foci, but over time each took on a wider range of activities and the merged organisation had hundreds of different services and products. As a result they were carrying a lot of cost and their purpose had become confused, both for staff and for customers. Also, their services were not used by the majority of potential customers. Before the transformation reported here, the charity’s strategy set an aspiration to reach more potential customers but it wasn’t designed to deliver the required step change. To address this, a change programme was initiated but it failed to get sufficient senior management sponsorship, and so the programme started as *skunk works* (a small group of people with autonomy to work on a “secret” project), led by the change management group, with no widespread communication and engaging only with those who had an appetite for change. This was to deliver a change programme for a new agile strategy that focused on a small number of activities. Hence, the change programme encompassed both organisational change and significant strategy change. The change manager, our gatekeeper, was keen to draw on previous Agile Transformation experiences and had self-trained in agile approaches and principles.

Prior to our involvement, an assessment of group culture and a re-structuring of the organisation had taken place. In particular there was an urgent need to improve the financial health of the organisation, and to embed the “lived experience” of disabled people into the organisation, by involving the community more. In April 2017, the organisation was restructured to remove duplicate functions, which resulted in the loss of senior management posts. From July 2017 a series of papers was put to the Board of Trustees setting out the development of a new strategy and delivery plan. Through these papers and ongoing work of the change management group, a new strategy evolved from then until its launch in late 2018.

Strategy development began with identifying an overarching vision and a set of ambitious goals. These were iterated through a group of 10–12 people invited to take part, from a range of different grades, departments, and physical locations around the country. The initial goals and objectives were tested with customer and internal staff stakeholders, and strategy drafts were regularly presented to the Board of Trustees.

Task and finish (T&F) groups were set up to drive the business plan forward. This included a subset of “Heads of Department”. Their remit included making sure that others in their department were kept informed of developments. The strategy development process aimed to produce a five-year and a three-year strategy, and a one-year plan. In the end, a five-year strategy, and a one year plan were delivered to the Board of Trustees, and the three-year plan was used as a basis for ongoing improvement.

## Method

We engaged with this case study from July 2017 to August 2018. The overall approach was to understand the transformation from the participants’ point of view rather than to impose any a priori expectations or analytical frameworks [9]. The initial meeting in July 2017 set the scene and agreed subsequent meetings. In Oct 2017 a workshop with the transformation team was held to explore agility and contextual matters including how to assess performance in an agile setting. Short catch-up phone calls (10–20 min) took place in Nov and Dec 2017, and longer interviews and discussions with members of the team (1–2 h) took place in January 2018 and March 2018. During these engagements, the team explained progress and shared their reflections. This data was used to construct a narrative of the transformation from the teams’ point of view.

In July 2018 we conducted semi-structured interviews with 9 Heads of Department about the transformation process to identify challenges, successes and next steps. These included Heads of Community Involvement, Customer Service, HR, Finance and Relationship Development. Our interviewees had been with the organisation for between 1 year and 17.5 years, and many were goal owners for the final strategy; none had received formal agile training. In August 2018 we conducted the same semi-structured interview with the Head of Transformation.

Throughout this time, researchers also had access to several documents and versions of the strategy including the one issued to staff in Sept 2018. This included a set of values and behaviours expected from staff and to be used as a guide for recruitment.

All interactive sessions were audio recorded and transcribed, or detailed notes were taken contemporaneously. The documentation, and some aspects of the audio recordings were used to construct the case background above. The views of the journey, including successes and challenges were analysed thematically [2].

## Results

We present the results from two perspectives: one focusing on the transformation team and the other focusing on the Department Heads. These two are compared in Sect. 5.

### Transformation from Inside the Agile Transformation Team^1^

The main engagements with the transformation team were in Oct 2017, and in January, March and August 2018. During **Oct 2017**, four related issues were discussed:

1. What is agility, and what is it not? Issues included the need for accountability, discipline, empowerment, customer focus, and responsiveness. Common misconceptions about agile that staff in the organisation may have were identified, including that agile isn’t chaotic or process-obsessed. A longer list of issues were identified for discussion later, including business readiness, appraisal of team and individuals, agile behaviours and consensus.
2. Performance management in an agile environment. For someone to be accountable, performance needs to be measured, but agile focuses on the team rather than the individual so how can performance of an individual rather than the team be measured?
3. Agile strategy. The strategy needs to be responsive to the environment and hence updated regularly. Discussion included the idea of a three-year rolling plan, and questions such as “where do I start?”, “what’s sprint 1?”, how to keep momentum going – not to just run workshops, get a brilliant “buzz” and then stall. An evidence base for challenging ideas and providing rapid feedback was needed.
4. Sustainability of agile. Agile behaviours and performance management were framed in terms of sustainability “*We can do agile planning, but agile sustainability comes down to what people are motivated to do…and how they are motivated to behave*”.

By **January 2018**, there was a sense in the transformation team that the process around the strategy needed to support its continuous improvement, and therefore should be agile. Although the original focus was on an agile strategy, they realised that “*Agile strategy has to be a process*”. The organisation had identified a long term vision, and developed a business plan with four priorities and eight cross-cutting objectives. The next step was to change the portfolio management process to adapt to having three-year rolling plans that move towards that long term goal, through three-month cycles to check progress “*is this the right stuff? Yes, move on; no, stop it or cut it*”. This will involve test-learn cycles. “*That’s your agile strategy, it’s your tactical 1*-*3 year business plan moving towards big significant goals, that get refined*”. Creating those business plans was underway, and a template for the business plan for each department had been developed. The culture change that was needed was planned to be driven through the new branding process, which was expected to launch towards the end of the year.

In **March 2018** (about half way through the transformation process), accountability continued to be a big issue, along with the need to identify and acquire appropriate data for performance management. Difficulties arose from senior managers concerned that they would be accountable for things outside their direct budgetary control. Some people associated accountability with blame, and were concerned about consequences if the objective failed. Although they were still not very agile, the changes so far highlighted the “*massive culture change required*”… “*fundamentally we are not currently built to deliver those goals*”. Instead of focusing on changing the culture, the terminology had changed to look at values and behaviours.

Fixed hierarchical structures and fixed timeframes were causing problems, and there was little appreciation that the plan had to drive activities. In the past, the plan was delivered through line management and the budget, and these are structured in silos. In a fixed governance structure it’s hard to explain the dynamic nature of the process.

A new operational model was being developed to offer more activities online. Staff and customers had been consulted about the plan and organisational changes; the goals and objectives had also been tested with customers and staff, including potential customers who had not engaged with the organisation before.

People were still working in silos creating their own plans, not talking across departments, and without reference to the overall goals – if it’s not in the strategy then “*you really shouldn’t be doing it…this isn’t about empowerment but discipline”.* Overall, the team felt “*it’s moving us in the right direction*” but “*we just assumed way too much*” and *“*<*the process*>* gently exposed some of the undercurrents of the organisation*”.

Highlights from the **Head of Transformation’s interview** in **August 2018** include:

“*the approach we’re moving towards is absolutely right* – *right for *<*the organisation*>* specifically but actually generically right for an awful lot of organisations*”… “*the change we’re seeing in our external customer environments is just not gonna stop*”

However, he also identified several challenges including

- Senior stakeholders may have buy-in to the process, but they also need to go through a personal change as well as a fundamental organisational transformation “*we didn’t appreciate the depth of mindset change basically that it would need*.”
- They needed more stability in terms of leadership
- Agility needs a collaborative way of working, which is counter to a hierarchical organisation with silos. “*half the senior management didn’t know what other functions did*” “*A key thing is just understanding what everyone does*”.
- Communicating the approach outside the managers involved was limited.
- Difficulty in communicating what accountability means – “*you may not be in control of all the direct levers for an outcome but you are in control of relationships with the people who can pull those levers*”
- Need a real-time (as close as possible) operational dashboard

Two main areas for improvement for the next cycle in the agile process are to:

- Be a bit more creative, e.g. using design thinking, so that people engage in real business change “*we need to focus a lot more on enabling the business change…and probably a bit less on the process itself*”
- Get new senior people up to speed quickly, or find a way to retain senior people. Constant change of personnel created instability.

In his view, strategic agility requires different perspectives to co-exist: a future vision that sets an aspiration, a medium term horizon: *“We now have the purpose statement and the priorities, and we have business plans, but we need to tackle the really important medium*-*term strategic goals.”*, and a short term horizon: *“our major Achilles heel across the whole charity is data … our new performance dashboard is a lot better … we’re nowhere near being able to report the real*-*time heartbeat type metrics that we really need to understand how the business is performing day*-*by*-*day”*

### Transformation from the Heads of Department Perspectives

The interviews with the Heads of Department were analysed for themes according to successes, challenges, what could have been done better in the transformation, and next steps. Table 1 summarises the themes emerging from this analysis. Note that the quotes do not represent the full data set; where cells are empty, no interviewee identified anything in that category, e.g. no-one suggested that aspects of Organisational Structure could have been Done Better.

**Table 1.** Themes from Heads of Department interviews, with illustrative quotes

Theme	Success	Challenge (past)	Challenge (future)	Done better	Next steps
Plan/strategy	“Strategy is great”	“No control or proper oversight”	“Succession planning strategy ownership”		
Org structure	“More manageable organisation”	“Organisation too convoluted”			
Org culture	“Shift in mentality”	“Honesty and openness”	“Morale”		
Org purpose	“Shared organisational goals”	“No guiding narrative or philosophy for decision-making”			“Be clear about Charity’s role”
Level of org change		“Degree of organisational change”	“Change fatigue”	“Stability, everything’s been changing”	“Get changes embedded”
Transformation process	“Process has been excellent”	“Process took too long”			
External profile	“Responding to external events well”	“Reputation declining for years”	“Need to make sure people know us”		“Launch ourselves as listening”
Operational	“Budget agreed”	“Identifying accountable owners”	“Data difficult to quantify”	“Find effective way to update finances”	“Articulate budget requirements”
Staff buy-in	“Getting senior people on the T&F group”	“People need to buy-in to the philosophy”	“Bring along people across the organisation”		“Excite and energise everyone”

Reading Table 1 left to right provides an overview of the theme and how it plays out across the transformation activities. For example (quotes come from different interviewees, so sentences do not represent any one person’s view):

#### Level of Organisational Change:

There was no mention of *success* in this theme. A *past* challenge was the high degree of organisational change, and a *future* challenge will be change fatigue. What could be *done better* is to achieve more stability as everything’s been changing, and *next steps* are to get the changes embedded.

#### External Profile:

A *success* was the response to external events. A *past* challenge was that reputation had been declining, and a *future* challenge will be to make sure people know what we stand for; *next steps* are to launch as a listening organisation.

## Discussion

The meaning of accountability was a concern for the transformation team throughout the process. It was mentioned in every engagement we had with the transformation team, but hardly mentioned at all in the Heads of Department interviews. Other issues raised by the transformation team were recognised by the Heads, but not all the issues raised by the Heads were recognised by the team.

There was a strong support for the progress that had been made up to the new strategy’s launch – not just the strategy itself, but also its vision and goals. Other successes related to the organisation’s structure, a change in culture and mindset, and the turnaround of the financial situation.

There were several past challenges, but fewer future challenges. Those that were identified relate to keeping staff engaged and energised in the continuing transformation process, succession planning for strategic development, getting the right data available to performance management, aligning the Departments and the strategic goals, and communicating the right external profile.

Areas for improvement in terms of the transformation process were maintaining more stability in the organisation, finding a better way to update finances, being clear and transparent in communications and expectations, and being more creative in how the process unfolds. The next steps identified were in response to the issues raised above, and included embedding changes, articulating clearly the organisation’s goals externally, and energising everyone to take the changes forward.

## Conclusion

*“I thought we’d embarked on achieving a destination, but actually what we embarked on was a really long journey”* Head of Transformation.

Strategic agility requires three different horizons to co-exist: a long term aspiration, a medium term set of goals, and a short-term response to real-time performance management. The experience of this case study shows that introducing this approach to a traditional, hierarchical organisation requires a number of conditions including: sufficient resources, stable leadership, and suitable performance measurement data.

Although not driven by IT or encompassing traditional Agile frameworks, this case study contributes empirical results to the growing set of transformation studies within the XP community. Future plans in this research include to engage with other organisations using a strategic approach to their transformation, and to compare these findings with organisations who take a different approach to transformation.

## Footnotes

1 All quotes in this sub-section are from a member of the transformation team (one of 3 people).
